# Impact of demographic factors on recognition of persons with depression and anxiety in primary care in Slovenia

**DOI:** 10.1186/1471-244X-8-96

**Published:** 2008-12-24

**Authors:** Janez Rifel, Igor Švab, Marija Petek Šter, Danica Rotar Pavlič, Michael King, Irwin Nazareth

**Affiliations:** 1 Department of family medicine, Medical faculty, University in Ljubljana, Slovenia; 2 Department of Mental Health Sciences, Royal Free and University College Medical School, University College London, Hampstead Campus, Rowland Hill Street, London NW3 2PF, UK; 3 General Practice Research Framework, University College London, Rowland Hill Street, London NW3 2PF, UK

## Abstract

**Background:**

Research has repeatedly shown that family physicians fail to diagnose up to 70% of patients with common mental disorders. Objective of the study is to investigate associations between persons' gender, age and educational level and detection of depression and anxiety by their family physicians.

**Methods:**

We compared the results of two independent observational studies that were performed at the same time on a representative sample of family medicine practice attendees in Slovenia. 10710 patients participated in Slovenian Cross-sectional survey and 1118 patients participated in a first round of a cohort study (PREDICT-D study). Logistic regression was used to examine the effects of age, gender and educational level on detection of depression and anxiety.

**Results:**

The prevalence of major depression and Other Anxiety Syndrome (OAS) amongst family practice attendees was low. The prevalence of Panic Syndrome (PS) was comparable to rates reported in the literature. A statistical model with merged data from both studies showed that it was over 15 times more likely for patients with ICD-10 criteria depression to be detected in PREDICT-D study as in SCS survey. In PREDICT-D study it was more likely for people with higher education to be diagnosed with ICD-10 criteria depression than in SCS survey.

**Conclusion:**

People with higher levels of education should probably be interviewed in a more standardized way to be recognised as having depression by Slovenian family physicians. This finding requires further validation.

## Background

Most people with depression are treated by their family physicians and only about 20% of people with the disorder are seen in secondary care [1]. On the other hand, research has repeatedly shown that family physicians fail to diagnose up to 70% of cases [1-3]. The proportion of cases missed varies according to the severity of depression with improved recognition by family physicians as the severity of illness increases [1,4,5].

Several studies have tried to identify factors that influence the recognition of mental disorders in primary care setting. Women [3,4,6], people with lower education [5], the elderly [4] and people who are separated, divorced or widowed [6] are more likely to be diagnosed with depression by their physicians. Other studies have found no influence of demographic factors on the detection of depression [7-9]. Coyne et al found no differences between those identified with major depression or not but when this was examined for broader defined depressive disorders (i.e. inclusive of dysthymias and adjustment disorders) younger people and those with education at the level of high school were less likely to be detected [1].

Population studies of 12-month prevalence of major depression in the USA report rates of 4,5% [10] and in the UK this is as high as 10% [11]. Associated adverse outcomes such as suicide and alcohol consumption differ across Europe with Slovenia being one of the ten European countries reporting the highest suicide rate and alcohol consumption [12,13].

Anxiety disorders are less well evaluated than depressive disorders but prevalence estimates indicate that one in four people experience one type of anxiety disorder in their life. The 12-month community prevalence of any type of anxiety disorder in the USA is 11,8% and the prevalence of generalized anxiety disorder and panic disorder was 2,8% and 1,4% respectively [10]. In primary care, the prevalence of general anxiety disorder is 8% and the prevalence of panic disorder is between 4–6% [14-16]. Little is known about the influence of demographic factors on recognition of anxiety disorders.

A recent study PREDICT-D reported prevalence of common mental disorders (major depression, other anxiety syndrome and panic disorder) in six different European countries [17]. Highest prevalence of disorders were found in the UK and Spain and lowest in Netherlands and Slovenia. Over all six countries prevalence of major depression, other anxiety syndrome and panic syndrome was 13,9%, 10,0% and 9,2% respectively for women and 8,5%, 5,0% and 5,6% respectively for men. In Slovenia prevalence of major depression, other anxiety syndrome and panic syndrome was 6,5%, 3,0% and 7,6% respectively for women and 4,4%, 2,2% and 4,7% respectively for men [17].

The aim of this paper is to investigate the demographic factors associated with the detection of depression and anxiety in family medicine practice attendees in Slovenia.

## Methods

This study was conducted in Slovenia, a central European country and member of the European Union since 2004 with a total population of 2 million. Data on a sample of family medicine practice attendees selected across the country was separately collected in two stages.

### The Slovenian Cross-sectional survey

Data from a random sample of 42 family medicine practitioners selected across the country was collected from October 2003 to March 2004 in the Slovenian Cross-sectional survey (SCS survey). Physicians were randomly selected from the register of currently active family medicine practitioners. Out of 850 physicians 50 were selected, 8 physicians didn't want to participate in the study. Physicians filled in a questionnaire for each patient attending or after every second or third attendee until they completed 300 questionnaires. Participating attendees gave oral informed consent for the participation in the study. Each physician chose his/her sampling strategy at the start and didn't change it during the course of the study. This was consistent for each physician and didn't change during the study. There were no exclusion criteria. Adapted NIVEL (Netherlands Institute for Health Services Research) questionnaire was used in SCS survey. The original NIVEL questionnaire contains questions about the date and place of the contact, patient's gender and birth year, problems presented by the patient, the aim of the contact, diagnostic procedures, preliminary diagnosis (maximum of three), therapeutic procedures, medicines prescribed and whether instructions were given on follow up. In the Slovenian version used in SCS survey, questions about the patient's education, change of general practitioner in the last year, type of contact, sick leave and consultation time were also obtained and the doctor was asked to list a maximum number of eight diagnoses. Family physicians were instructed to report all the patient's diagnoses of mental or physical diseases or disorders physicians were aware of, no matter if the diagnose was recorded in the medical file or not. The diagnoses were coded by trained resident doctors into International Classification of Primary Care codes – second edition (ICPC-2) [18]. Code P74 from ICPC-2 relates to F41.0, F41.1, F41.3 to F41.9 codes of the International Classification of Diseases (ICD-10). Code P76 from ICPC-2 relates to F32, F33, F34.1, F34.8, F34.9, F38, F39, F41.2 and F53.0 codes of the ICD-10.

National Medical Ethics Committee of the Republic of Slovenia approved the protocol of the SCS survey.

### The PREDICT-D study

The PREDICT-D study is the first large scale study in Slovenia measuring prevalence of common mental disorders in primary care settings using standardized diagnostic questionnaires on a representative sample of family medicine practice attendees. Consecutive family medicine practice attendees aged 18 to 75 years were recruited and followed up after six, 12 and 24 months. The study design has been previously described [19]. The aim of the PREDICT-D study was to develop a reliable and valid multi-factor scale to determine the risk for the onset and maintenance of depression in primary care attendees. The participating family medicine practices were selected from urban and rural settings in each country and served a population with diverse socio-economic and ethnic characteristics. In Slovenia the study was conducted across 74 family medicine practices nationwide. Each practice recruited 10–20 participants. Each participant signed written informed consent for the participation in the study at baseline. Baseline interviews were carried out between September 2003 and March 2004 by 36 trained interviewers who were mostly medical students. Mood was examined using the Depression Section of the Composite International Diagnostic Interview (CIDI)[20,21], which provided psychiatric diagnoses based on symptoms experienced in the last six months according to Diagnostic and Statistical Manual of Mental Disorders (DSM-IV) criteria and ICD-10 criteria. Anxiety disorders were examined using the Patient Health Questionnaire (PHQ) [22], a brief questionnaire designed to assess DSM-IV Other Anxiety Syndrome (OAS) and Panic Syndrome (PS). Information on socio-demographic characteristics including gender, age and educational level of the participants was also collected using a standardised questionnaire for this purpose [19]. All questionnaires were in Slovene language. Exclusion criteria were inability to understand Slovene language, severe organic mental illness and terminal illness. Slovene language version of CIDI was psychometrically validated before the study but the validation process was not published. Slovenian version of PHQ was not psychometrically validated before the study.

National Medical Ethics Committee of the Republic of Slovenia approved the protocol of the PREDICT-D study.

### Reducing possible bias

Data was collected by several people in both studies. In the PREDICT-D study researchers were trained to minimize interviewer bias. Moreover, the diagnosis of depression or anxiety disorder could be perceived as stigmatising for patients and to allow patients to provide an honest account of their symptoms we made it known to them that this information would not be passed on to their practice staff. However, they were informed that the interviewer would contact their family physician if he/she were worried about their safety (e.g. suicidal plans). In the SCS study practically no patients refused participation but in the PREDICT-D study 20% refused to participate.

### Similarities and differences between studies

Both studies sampled primary care attendees over the same time period. The essential difference between the two studies was that in the first family physicians were asked to note all the diagnoses of the participants, which included depression and/or anxiety, and in the second study diagnoses of depression and anxiety disorders were ascertained using standardised diagnostic interviews by research interviewers. Both studies report cross-sectional data. The PREDICT-D study was a part of a larger prospective study done in six European countries to develop a valid and reliable instrument to predict future episodes of depression. In this paper we report the baseline Slovenian data.

### Statistical analysis

Data were analysed using SPSS for Windows version 16. Logistic regression was used to determine the effect of demographic factors on detection of depression and anxiety separately in both datasets.

## Results

In SCS survey data of 12596 patient contacts were collected. The patients varied in age from 0 to 98 years. We restricted our analysis to 10701 people aged 18–75 as this was the age range recruited to the PREDICT-D study.

In PREDICT-D study 1121 patients took part. Three patients were excluded as they were not within the age range of 18 – 75 years. 276 of those approached to take part refused to participate in the study.

There were significant differences in age, gender and education between people included in the SCS survey and PREDICT-D study (See Table 1). The prevalences of depression and anxiety are presented in Table 2. In the PREDICT-D study approximately twice as many patients were found to have DSM IV major depression and five times as many patients had depression according to ICD-10 criteria when compared with those diagnosed by the family doctor in the SCS survey. The PREDICT-D study reported a seven times greater likelihood of panic syndrome and/or other anxiety syndrome than the SCS survey.

**Table 1:** Demographic characteristics of samples in SCS survey and PREDICT-D study.

	**SCS survey (n = 10710)**	**PREDICT-D study (n = 1118)**
Mean age (years)	49.67 (SD = 15,23)	48.73 * (SD = 14,42)
Female (percent)	53.3	63.4 **
Educational level (percent):		**
Primary	36.3	22.3
Professional	26.4	23.4
Secondary	25.2	37.4
Higher	5.8	6.9
University	6.4	10.0

* p < 0.05, ** p < 0.0001

**Table 2:** Prevalence of depression and anxiety in SCS survey and PREDICT-D study in percents, numbers of persons in parenthesis; PS-panic syndrome, OAS-other anxiety syndrome.

		**Depression**		**Anxiety**		
	
**SCS survey (n = 10710)**	Women	3.8 (217)		1.4 (77)		
	Men	1.8 (91)		0.8 (41)		
	Total	2.9 (308)		1.1 (118)		
		**Major depression**	**ICD-10 criteria depression**	**PS**	**OAS**	**PS and/or OAS**
	
PREDICT-D study (n = 1118)	Women	6.5** (46)	16.4** (116)	7.6** (54)	3.0** (21)	9.2** (64)
	Men	4.4** (18)	11.2** (46)	4.7** (19)	2.2* (9)	6.4** (26)
	Total	5.8** (64)	14.5** (162)	6.5** (73)	2.7** (30)	8.2** (90)

* p < 0.01, ** p < 0.001

Table 3 lists the results of the multivariable analyses on the SCS survey to examine the association of age, gender and educational level on the recognition of patients with depression and anxiety. These results are presented as odds ratios and 95% confidence intervals.

**Table 3:** Odds ratios for effect of sociodemographic factors on detection of depression and anxiety in SCS survey (n = 10450).

	**Depression (n = 300)**	**Anxiety (n = 112)**
	OR (95% CI)	OR (95% CI)
Gender (woman = 0, man = 1)	0.46*** (0.36–0.60)	0.56** (0.38–0.84)
Age	1.00 (0.99–1.00)	0.99 (0.98–1.01)
Educational level		
Primary	1.00 (n = 137)	1.00 (n = 40)
Professional	1.00 (0.75–1.32) (n = 86)	1.13 (0.69–1.84) (n = 30)
Secondary	0.60** (0.43–0.83) (n = 59)	1.09 (0.67–1.79) (n = 32)
Higher	0.47* (0.25–0.87) (n = 11)	0.72 (0.28–1.86) (n = 5)
University	0.28** (0.13–0.60) (n = 7)	0.67 (0.26–1.73) (n = 5)

* p < 0.05; **p < 0.01; ***p < 0.001

Depression was significantly more likely to be identified in women and those less well educated whereas anxiety was more likely to be identified in women.

Table 4 provides the results of the multi-variable analyses conducted on the PREDICT-D dataset. Women demonstrated at least a 1.5–2.00 time greater prevalence of ICD-10 depression and panic syndrome and university educated people were 5.6–11.1 time less likely to have panic syndrome and/or only other anxiety syndrome or only panic syndrome respectively than people with primary education.

**Table 4:** Odds ratios for effect of sociodemographic factors on presence of mental disorders in PREDICT-D study (n = 1116)

	**Major depression** **(n = 64)**	**ICD-10 depression** **(n = 162)**	**Panic disorder** **(n = 73)**	**PS and/or OAS** **(n = 90)**
	OR (95% CI)	OR (95% CI)	OR (95% CI)	OR (95% CI)
Gender (woman = 0, man = 1)	0.65 (0.37–1.14)	0.65* (0.45–0.94)	0.52* (0.30–0.91)	0.62 (0.38–1.00)
Age	1.00 (0.98–1.01)	0.99 (0.98–1.00)	1.00 (0.98–1.02)	1.01 (0.99–1.02)
Educational level				
Primary (reference category)	1.00 (n = 12)	1.00 (n = 33)	1.00 (n = 22)	1.00 (n = 25)
Professional	1.18 (0.53–2.63) (n = 14)	1.04 (0.62–1.76) (n = 35)	1.22 (0.66–2.27) (n = 25)	1.26 (0.71–2.26) (n = 29)
Secondary	1.59 (0.79–3.20) (n = 32)	1.23 (0.77–1.95) (n = 70)	0.58 (0.31–1.08) (n = 22)	0.77 (0.43–1.35) (n = 31)
Higher	1.06 (0.33–3.41) (n = 4)	0.86 (0.39–1.90) (n = 9)	0.42 (0.12–1.45) (n = 3)	0.38 (0.11–1.29) (n = 3)
University	0.34 (0.72–1.55) (n = 2)	0.88 (0.45–1.74) (n = 15)	0.09* (0.01–0.68) (n = 1)	0.18* (0.04–0.80) (n = 2)

* p < 0,05

We did not run an analysis on the detection of OAS as there were insufficient numbers (n = 30) and none with OAS and higher education.

**Figure 1:**
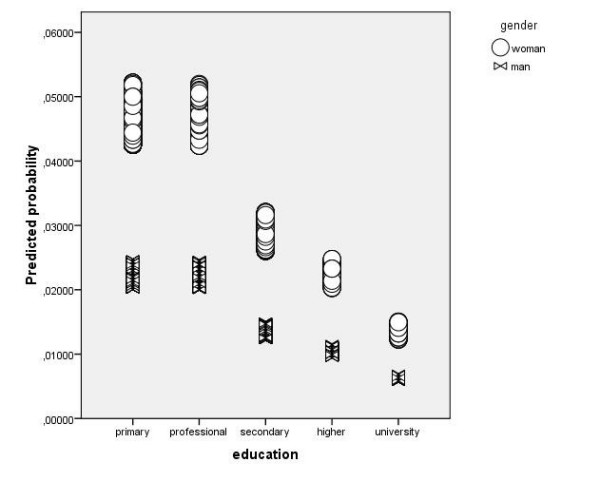
**Predicted probability for ICD-10 defined depression for the level of education only for SCS survey cases (n = 10450)**.

**Figure 2:**
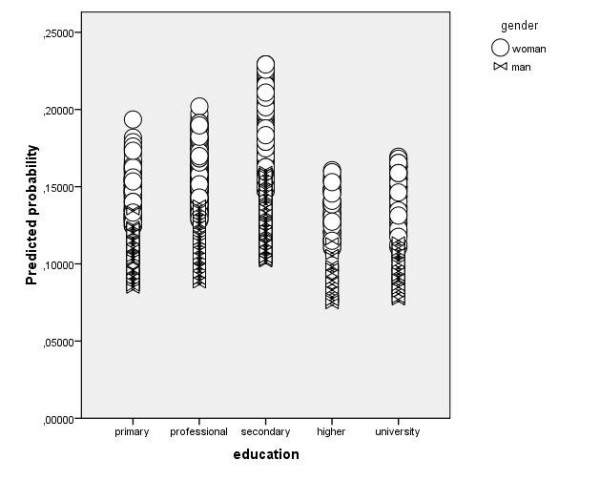
**Predicted probability for ICD-10 defined depression for the level of education only for PREDICT-D study cases (n = 1116)**.

To take into account the differences between the demographic structures of the two studies and compare the effect of covariates on the disorder detection, logistic regression analysis allowing for interaction terms was performed on a merged data set. Analyses of interactions showed that there were no significant differences between diagnosed patients with depression in SCS and patients with major depression in PREDICT-D study for gender, age or educational level. But in the case of depression defined by ICD-10 criteria analyse showed that interaction between study and educational level was significant. Interactions for gender and age were non-significant. In PREDICT-D study it was more likely for people with higher education to be diagnosed with depression than in SCS survey. Overall it was over 15 times more likely for patients with ICD-10 criteria depression to be detected in PREDICT-D study as in SCS survey. Results of logistic regression analysis allowing for interaction terms for the case of depression defined by ICD-10 criteria are showed in Table 5 and Figures 1 and 2.

**Table 5:** Results of the logistic regression analysis allowing for interaction terms in the case of depression defined by ICD-10 criteria

	**df**	**Sig.**	**Odds Ratio**	**95,0% C.I. for Odds Ratio**	
				Lower	Upper
gender	1	,000	,461	,357	,595
age	1	,361	,996	,988	1,004
education	4	,000			
education(1)	1	,980	,996	,750	1,324
education(2)	1	,002	,602	,434	,834
education(3)	1	,016	,465	,249	,870
education(4)	1	,001	,279	,129	,604
study	1	,000	15,678	5,212	47,158
age × study	1	,423	,994	,980	1,009
gender × study	1	,143	1,400	,892	2,198
education × study	4	,025			
education × study(1)	1	,028	,315	,113	,882
education × study(2)	1	,034	,331	,119	,920
education × study(3)	1	,385	,643	,238	1,740
education × study(4)	1	,421	,584	,158	2,163
Constant	1	,000	,059		

(gender: 0 = woman, 1 = man; age in years; education: reference category is primary education; research: 0 = SCS survey, 1 = PREDICT-D study) n = 11566.

Figures 1 and 2 show three things:

a) probability for depression is more or less equal across different levels of education for PREDICT-D study and is on the other hand decreasing with the level of education for SCS survey. In table 5 it is shown that interaction between education and study is statistically significant.

b) on both figures scatter plots are stratified for gender and in both groups women have higher probability for depression although this difference is more prominent in the SCS survey. This is true for every one of the five levels of education. This interaction between gender and study is not statistically significant though (Table 5)

c) Finally probability to predict depression is higher for the cases in PREDICT-D study than for SCS survey. This is logical as prevalence of detected patients with ICD-10 criteria in PREDICT-D study is five times higher as prevalence of detected patients with depression in SCS survey. Nevertheless mean predicted probability for depression in PREDICT-D study is only around 0,15.

## Discussion

### Impact of demographic factors on recognition of common mental disorders

Patients with higher education were more likely to be diagnosed with a standardized interview. Age and gender had no such effect. A logistic model based on merged data from both datasets showed significant effect of educational level in case of ICD-10 depression but no significant effect in case of major depression or anxiety. Educational level was strongly associated with recognition of depression in SCS survey and yet in the PREDICT-D study the prevalence of DSM IV major depression or ICD-10 depression had no relationship to educational status.

In both studies women were more likely to be diagnosed by the doctors and to have prevalent mental disorders. Similar results have been previously demonstrated in other studies, where primary care physicians detected only a minority of the psychiatric cases and the undetected cases belonged more commonly to the highest social groups [1,5].

Predicted probabilities for depression in regression model were low. It should be noted that our regression model was not designed to predict probability of depression but to investigate associations of three demographic factors with different modes of detection of patients with depression and/or anxiety.

### Prevalence of common mental disorders in primary care in Slovenia

The PREDICT-D study is the first large scale study in Slovenia measuring prevalence of common mental disorders in primary care settings using standardized diagnostic questionnaires on a representative sample of family medicine practice attendees. We found a surprisingly low prevalence of major depression (5,8%), especially considering the high rate of suicide and alcohol consumption previously reported in Slovenia [12,13]. We also found a low prevalence of Other Anxiety Syndromes of 2,7%, while the prevalence of Panic Syndrome of 6,5% that is comparable to the rates reported in literature [14-16].

PREDICT-D study found significant variations between European nations with Slovenian sample showing lowest prevalence [17]. It is possible that there are differences in consulting behaviour by people with mental disorders in different European countries.

This paper identifies differences in detection between patients during routine work in family medicine practices and patients assessed using standardized diagnostic questionnaires. Prevalence of DSM IV major depression was approximately twice as high as that detected by family doctors and this increased fivefold when applied to all categories of ICD-10 depression (i.e. mild, moderate and severe depression). Similar lower levels of detection of panic syndrome and other anxiety disorders were also observed when comparing data from the two studies. It is possible that people not detected with depression in primary care have milder depression than those detected [1]. Also CIDI instrument has a high sensitivity, but only moderate specificity. We detected also some healthy individuals as false positive cases of depression in PREDICT-D study, which exaggerates differences in prevalence between two studies.

### Strengths and limitations of the studies and comparative analysis

A major strength of both studies is the large sample of participants and family physicians. In SCS survey patients with mental disorders were diagnosed by the family physicians during routine consultations. Family physicians were not specifically asked to include only mental or behavioural diagnoses but all medical diagnoses they were aware of. In PREDICT-D mental disorders were ascertained using standardised questionnaires. The main limitation of this paper is that these data were not collected on the same attendees as we did not have the resources to run a similar SCS survey on the PREDICT-D study population. Samples, sites and recruitment methods varied to a certain extent between the studies. Comparability between the studies is thus very limited and our conclusions should be interpreted with caution. Our study can not be termed as a study on detection. Amalysis showed interaction between study and educational level. Study may stand for method of detection but as the samples differed there may as well be other influential factors that were not assessed.

The effect of practice clustering on detection of mental disorders was not introduced in our model although effect of family physicians could be added as a random effect. We could not estimate such models with our software. The study population in the SCS survey were marginally older, with fewer women and well educated people than those recruited to the PREDICT-D study. There were only few people who refused participation in SCS survey. In PREDICT-D study response rate was 80%. There were no significant differences in gender and age between participants and non-participants in PREDICT-D study, we don't have the data on educational level of non-participants in PREDICT-D study. We can only speculate that differences in educational level could be the consequence of recruitment and participation. Despite these limitations, it is possible that the differences between two studies were not substantial as the response rates were very high in both studies and they were both conducted at the same time with an overlap in the family practices that took part.

## Conclusion

Our data show that family physicians in Slovenia may under diagnose better educated patients with depression. Higher educated patients should be interviewed in a more standardized way in order to get to a diagnosis of depression. There is a need to explore the reasons underlying this reduced detection rate and to gain an understanding of the training and developmental agenda for family doctors working in Slovenian family medicine.

## Competing interests

The authors declare that they have no competing interests.

## Authors' contributions

JR led on writing the paper and analysed data. IŠ and IN originated the idea for the paper and revised the drafts of the paper. MPŠ and DRP coordinated PREDICT-D and SCS studies in Slovenia. MK and IN originated the idea for the PREDICT-D study, led on its design, obtained funding and coordinated the project. All authors read and approved the final manuscript.

## Pre-publication history

The pre-publication history for this paper can be accessed here:


